# Identification of novel genes associated with a poor prognosis in pancreatic ductal adenocarcinoma via a bioinformatics analysis

**DOI:** 10.1042/BSR20190625

**Published:** 2019-08-02

**Authors:** Jun Zhou, Xiaoliang Hui, Ying Mao, Liya Fan

**Affiliations:** 1 Department of General Ward 1, Zhejiang Hospital of Lingyin District, Zhejiang, China; 2 Department of Gastroenterology, Zhejiang Hospital of Sandun District, Zhejiang, China

**Keywords:** Carcinoma, MicroRNAs, Molecular Targeted Therapy, Pancreatic Ductal

## Abstract

Pancreatic ductal adenocarcinoma (PDAC) is a class of the commonest malignant carcinomas. The present study aimed to elucidate the potential biomarker and prognostic targets in PDAC. The array data of GSE41368, GSE43795, GSE55643, and GSE41369 were downloaded from Gene Expression Omnibus (GEO) database. The differentially expressed genes (DEGs) and differentially expressed microRNAs (DEmiRNAs) in PDAC were obtained by using GEO2R, and overlapped DEGs were acquired with Venn Diagrams. Functional enrichment analysis of overlapped DEGs and DEmiRNAs was conducted with Metascape and FunRich, respectively. The protein–protein interaction (PPI) network of overlapped DEGs was constructed by STRING and visualized with Cytoscape. Overall survival (OS) of DEmiRNAs and hub genes were investigated by Kaplan–Meier (KM) plotter (KM plotter). Transcriptional data and correlation analyses among hub genes were verified through GEPIA and Human Protein Atlas (HPA). Additionally, miRNA targets were searched using miRTarBase, then miRNA–DEG regulatory network was visualized with Cytoscape. A total of 32 DEmiRNAs and 150 overlapped DEGs were identified, and Metascape showed that DEGs were significantly enriched in cellular chemical homeostasis and pathways in cancer, while DEmiRNAs were mainly enriched in signal transduction and Glypican pathway. Moreover, seven hub genes with a high degree, namely, V-myc avian myelocytomatosis viral oncogene homolog (MYC), solute carrier family 2 member 1 (SLC2A1), PKM, plasminogen activator, urokinase (PLAU), peroxisome proliferator activated receptor γ (PPARG), MET proto-oncogene, receptor tyrosine kinase (MET), and integrin subunit α 3 (ITGA3), were identified and found to be up-regulated between PDAC and normal tissues. miR-135b, miR-221, miR-21, miR-27a, miR-199b-5p, miR-143, miR-196a, miR-655, miR-455-3p, miR-744 and hub genes predicted poor OS of PDAC. An integrative bioinformatics analysis identified several hub genes that may serve as potential biomarkers or targets for early diagnosis and precision target treatment of PDAC.

## Introduction

Pancreatic ductal adenocarcinoma (PDAC) is the most common pancreatic neoplasm, accounting for approximately 90% of all pancreatic cancers (PCs), with a poor overall survival (OS) rate of 5–8% [1,2]. Over 52% of cases are diagnosed in a distal metastasis stage, with only 3% showing a 5-year OS benefit [3]. Currently, surgical extirpation is the primary curative strategy; however, most patients with PDAC do not display any specific early signs of clinical disease, which causes missed opportunities for surgery due to the progression of cancer without detection. The preferred and most promising strategy for PC treatment is surgical resection, and the detection of PC during the surgically resectable stages has vast importance for ameliorating the survival benefit of patients with PC [4]. Therefore, an exact, early diagnosis of PDAC is needed, and the discovery of molecular biomarkers and targets could be another promising strategy to promote further developments in therapeutic modalities and strategies.

Various factors are associated with the growth and development of cancer and PDAC, including mutation, age, sex, ethnicity, cigarette smoking, and viral and bacterial infections [5–9]. The association of infectious agents in the etiology of different types of cancer has piqued the interest of scientists in recent years. Recent data have shown the involvement of *Mycoplasma hominis, Chlamydia pneumoniae*, and *Escherichia coli* infection in the etiology of prostate cancer, lung cancer, and colon cancer, respectively [9–12]. Recently, a wealth of previous studies have reported that microRNAs (miRNAs), a class of noncoding RNAs characterized by approximately 22 nucleotides in length, can inhibit the stability and translation of messenger RNAs (mRNAs) through binding to the specific sequence of genes, which are used as potential biomarkers for different kinds of cancer. Su et al. (2018) [13] employed Weighted Gene Co-Expression Network Analysis (WGCNA) to analyze 88 patients with PDAC and 19 healthy controls and reported that five miRNAs (miR-3201, miR-890, miR-16-2-3p, miR-877, and miR-602) were defined as promising diagnostic and prognostic biomarkers for PDAC. Additionally, miR-661 is up-regulated in PDAC and simultaneously promotes the protein expression level of transcription factor 4, β-catenin, and cyclin D1, which are associated with the Wnt signaling pathway *in vitro*, and can be used as a prognostic marker in suspicious pancreatic lesions [14]. Wei et al. (2018) [15] demonstrated that the overexpression of miR-23b-3p decreased tumor volume or even prevented the formation of tumors by directly down-regulating annexin A2 (ANXA2). Many biomarkers and therapeutic targets play a vital role in the diagnosis and treatment of PDAC. However, no breakthrough in the conventional treatment strategy has been investigated because of the difficulty in the early diagnosis and ominous prognosis of PDAC, and there is urgent need to identify more genetic information about pivotal genes in the progression of this disease.

Several *in silico* bioinformatics tools are widely applied to assess gene expression levels, to screen unique genes from RNA-sequencing and next-generation sequencing data, and to explore their possible implication in the growth of different types of cancer [16–18]. Currently, microarrays have been widely employed to detect genetic alterations during the initiation and progression of tumorigenesis. Accumulating data on malignancy are available at the authoritative Gene Expression Omnibus (GEO) (http://www.ncbi.nlm.nih.gov/geo/) genomics data repository that includes microarray and high-throughput sequencing data [19]. In the present paper, we employed mRNA microarray data of GSE41368 [20], GSE43795 [21], and GSE55643 [22] and miRNA microarray data of GSE41369 [20] to identify the differentially expressed genes (DEGs) and differentially expressed miRNAs (DEmiRNAs) and to explore the potential therapeutic targets as well as the prognostic value of PDAC. Compared with a single expression profile, the bioinformatics analysis of the overlapping DEGs in more chips could be more reliable. In the current study, we performed a comprehensive bioinformatics analysis to identify hub overlapping DEGs, and GO terms and KEGG pathway enrichment analyses of the DEGs and DEmiRNAs were performed. A protein–protein interaction (PPI) network and a DEmiRNA target network were constructed to assess the interactions between the DEmiRNAs and hub genes. Moreover, seven significant hub genes and ten DEmiRNAs were revealed to be associated with OS in PDAC by the Kaplan–Meier (KM) plotter (KM plotter). In addition, the mRNA expression level, correlation analysis and protein expression of the hub genes were validated with the GEPIA and Human Protein Atlas (HPA) databases. The current research aimed to explore potential hub genes that may be highly correlated with the prognostic value of PDAC from a multicultural perspective with systems biology. An integrated analysis of the DEGs in PDAC will provide further insight into the mechanism of PDAC.

## Materials and methods

### PDAC dataset preprocessing

The gene expression profile data (GSE41368, GSE43795, GSE55643, and GSE41369) of PDAC were downloaded from the public GEO database by searching with the following key terms: (‘microRNA’ OR ‘miRNA’ OR ‘mRNA’) AND (‘pancreatic ductal adenocarcinoma’ OR ‘PDAC’). The publication time was not limited. All included chip datasets have the same characteristics, that is, the comparison of human PDAC and normal specimens, and included at least six corresponding samples as well as all the microarray data available to the public.

### Data processing

GEO2R (https://www.ncbi.nlm.nih.gov/geo/geo2r/) is an interactive online tool that can compare different groups in a GEO series and can be employed to identify DEGs and DEmiRNAs. Subsequently, by default, the adjusted *P*-values were selected to decrease the false-positive rate using the Hochberg false discovery rate and the Benjamini method. A *P-*value <0.05 and an absolute log fold-change (FC) greater than 1 for the DEGs and 2 for the DEmiRNAs were used as the cut-off criteria. We also used the online tool Venny 2.1 (http://bioinfogp.cnb.csic.es/tools/venny/index.html) to identify the overlapping DEGs that were up-/down-regulated in the GSE41368, GSE43795, and GSE55643 datasets.

### Functional enrichment analysis of the overlapping DEGs and DEmiRNAs

GO terms and KEGG (Kyoto Encyclopedia of Genes and Genomes) pathway enrichment analyses play a vital role in identifying characteristic biological attributes for high-throughput transcriptome data. We used Metascape (http://metascape.org/), a gene annotation, and analysis resource [23], to perform a functional enrichment analysis, which included cellular component (CC), molecular function (MF), and biological process (BP), and a KEGG pathway analysis of the overlapping DEGs. Similarly, we also performed a functional enrichment analysis of the DEmiRNAs using FunRich, which is also primarily used for the interaction network and the functional enrichment analysis of genes and proteins [24]. Bubble diagrams and bar charts, respectively, were used to visualize the results of the GO terms and KEGG pathway enrichment analyses of the overlapping DEGs and DEmiRNAs.

### Construction of the PPI network of the overlapping DEGs

STRING (https://string-db.org/) is a user-friendly online system that provides predicted and experimental interactions of proteins [25]. We used the STRING database to analyze functional interactions between the overlapping DEGs with a confidence score of ≥0.4 for significant differences. The PPI network was visualized with Cytoscape (version 3.6.0, www.cytoscape.org), and the properties of the network topology for nodes were calculated to identify hub genes with a degree >5 in the present study. The degree indicates the number of edges connected with a specific node, and nodes with a high degree were identified as hub genes (i.e., may contribute to vital biological behaviors).

### Survival analysis of the DEmiRNAs and hub genes

We used the KM plotter (http://kmplot.com/analysis/) [26] to perform an OS analysis of the DEmiRNAs and hub genes in 178 and 177 patients with PDAC, respectively. The plotter endows users with the ability to separate patients divided into high and low expression groups on the basis of the gene transcriptional expression level of a given gene and create KM plots. In addition, the hazard ratio (HR) with the 95% confidence interval and the log-rank *P*-value were calculated and are shown on the chart, and the number-at-risk is displayed below the curves.

### Expression levels and correlation analysis of the hub genes

GEPIA (Gene Expression Profiling Interactive Analysis) is a well-developed interactive online server applied to a standard processing pipeline that analyzes the RNA sequencing expression data of 9736 tumors and 8587 normal samples from The Cancer Genome Atlas (TCGA) and the Genotype-Tissue Expression (GTEx) projects [27]. We used GEPIA to investigate the mRNA expression levels of hub genes in PDAC and normal tissues. Then, a boxplot was generated to show the relation. The HPA (https://www.proteinatlas.org/) is an open access to enable scientists in both industry and academia to freely access data for exploration of the human proteome [28]. The HPA database was also used to validate the immunohistochemistry of the candidate hub genes. The direct link to these images in the HPA are as follows: https://www.proteinatlas.org/ENSG00000136997-MYC/tissue/pancreas#img (v-myc avian myelocytomatosis viral oncogene homolog, MYC, in normal tissue); https://www.proteinatlas.org/ENSG00000136997-MYC/pathology/tissue/pancreatic+cancer#img (MYC in tumor tissue); https://www.proteinatlas.org/ENSG00000117394-SLC2A1/tissue/pancreas#img (solute carrier family 2 member 1, SLC2A1, in normal tissue); https://www.proteinatlas.org/ENSG00000117394-SLC2A1/pathology/tissue/pancreatic+cancer#img (SLC2A1 in tumor tissue); https://www.proteinatlas.org/ENSG00000067225-PKM/tissue/pancreas#img (Pyruvate kinase muscle isozymes, PKM, in normal tissue); https://www.proteinatlas.org/ENSG00000067225-PKM/pathology/tissue/pancreatic+cancer#img (PKM in tumor tissue); https://www.proteinatlas.org/ENSG00000122861-PLAU/tissue/pancreas#img (plasminogen activator, urokinase, PLAU, in normal tissue); https://www.proteinatlas.org/ENSG00000132170-PPARG/tissue/pancreas#img (peroxisome proliferator activated receptor (PPAR) γ, PPARG, in normal tissue); https://www.proteinatlas.org/ENSG00000132170-PPARG/pathology/tissue/pancreatic+cancer#img (PPARG in tumor tissue); https://www.proteinatlas.org/ENSG00000105976-MET/tissue/pancreas#img (MET proto-oncogene, receptor tyrosine kinase, MET, in normal tissue); https://www.proteinatlas.org/ENSG00000105976-MET/pathology/tissue/pancreatic+cancer#img (MET in tumor tissue); https://www.proteinatlas.org/ENSG00000005884-ITGA3/tissue/pancreas#img (integrin subunit α 3, ITGA3, in normal tissue); and https://www.proteinatlas.org/ENSG00000005884-ITGA3/pathology/tissue/pancreatic+cancer#img (ITGA3 in tumor tissue). A correlation analysis presents pairwise genes using the Pearson, Spearman, and Kendall correlation statistics based on GTEx and/or TCGA expression data in GEPIA [18].

### Construction of the miRNA–mRNA network

The genes targeted by the DEmiRNAs were predicted using miRTarBase (http://miRTarBase.mbc.nctu.edu.tw/), the experimentally validated miRNA–target interactions database [29]. Next, the regulatory network of the predicted genes and miRNAs that targeted them were visualized in Cytoscape.

## Results

### Identification of the DEGs and DEmiRNAs

**Figure 1:**
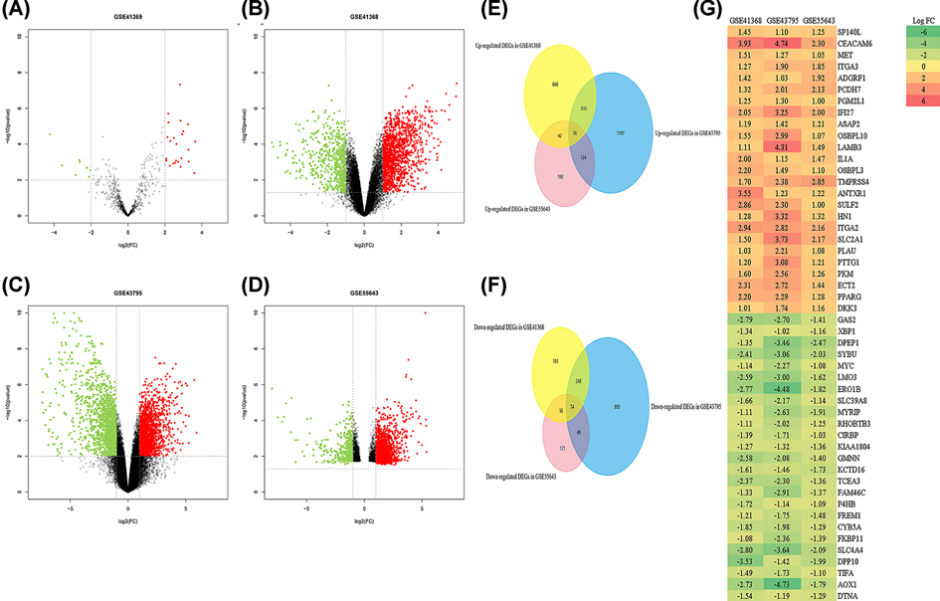
Volcano plot of gene expression profile data in PDAC and normal samples and the heatmap of the overlapping DEGs (**A**) Volcano plot of GSE41369, (**B**) volcano plot of GSE41368, (**C**) volcano plot of GSE43795, (**D**) volcano plot of GSE55643, (**E**) Venn diagram of the up-regulated overlapping DEGs, (**F**) Venn diagram of the down-regulated overlapping DEGs, and (**G**) heatmap of the overlapping DEGs. Green represents a low FC value, and red represents a high FC value. Each column represents one dataset, and each row represents one gene. The number in each rectangle represents the FC in PDAC samples compared with normal samples. The gradual color change from red to green represents the changing process from up-regulation to down-regulation.

The characteristics of the available profiles are presented and include the PubMed ID, GEO accession number, experiment type, sample source, number of tumors and controls, platforms, corresponding author, and publication time (Table 1). As shown in Table 1, 57 PDAC samples and 20 normal samples were applied for RNA-seq analysis, and 9 PDAC samples and 9 normal samples were employed for miRNA-seq analysis. For the GSE41369 dataset, a total of 32 DEmiRNAs (24 up-regulated and 8 down-regulated miRNAs, Figure 1A and Table 2) were extracted, and a total of 1828, 3233, and 1144 genes were extracted from the GSE41368, GSE43795, and GSE55643 datasets, respectively (Figure 1B–D); these miRNAs and genes were recognized as differentially expressed in PDAC samples compared with normal samples. Among them, 150 DEGs overlapped; 76 were up-regulated (Figure 1E), and 74 were down-regulated (Figure 1F). Additionally, based on the logFC value, we show the top 25 up- and down-regulated DEGs as a heat map in Figure 1G.

**Table 1:** The data characteristics of the gene expression profiling studies

PMID	Record	mRNA/miRNA	Tissue	Normal	Tumor	Platfrom	Reference
24120476	GSE41368	mRNA	PDAC	6	6	GPL6244-[HuGene-1_0-st]Affymetrix Human Gene 1.0 ST Array [transcript (gene) version]	Frampton et al., 2014
24072181	GSE43795	mRNA	PDAC	6	6	GPL10558-Illumina HumanHT-12 V4.0 expression beadchip	Kim et al., 2014
25415223	GSE55643	mRNA	PDAC	8	45	GPL6480 Agilent-014850 Whole Human Genome Microarray 4x44K G4112F (Probe Name version)	Jamieson et al., 2014
24120476	GSE41369	miRNA	PDAC	9	9	GPL16142 NanoString nCounter Human miRNA assay (v1)	Frampton et al., 2014

**Table 2:** The up-/down-regulated DEmiRNAs in GSE41369

Up/down	DEmiRNAs	*adj.P.Val*	*P*-value	logFC
Up-regulated	hsa-miR-135b	0.0000337	4.63E-08	2.819987
	hsa-miR-221	0.0007128	1.96E-06	2.181648
	hsa-miR-10a	0.0011033	4.87E-06	2.841119
	hsa-miR-197	0.0011033	6.07E-06	2.192276
	hsa-miR-145	0.0011327	7.79E-06	3.244704
	hsa-miR-21	0.0019778	1.90E-05	2.992536
	hsa-miR-223	0.0020383	2.80E-05	2.930734
	hsa-miR-342-3p	0.0024343	4.02E-05	2.136132
	hsa-miR-125a-5p	0.002574	4.60E-05	2.483418
	hsa-miR-1975	0.0034239	6.74E-05	2.291544
	hsa-miR-27a	0.0034239	7.06E-05	3.636233
	hsa-miR-142-5p	0.0039221	9.87E-05	2.625738
	hsa-miR-199a-5p	0.0070605	2.04E-04	3.264147
	hsa-miR-142-3p	0.0159969	6.14E-04	2.892267
	hsa-miR-129-3p	0.0159969	6.56E-04	2.073776
	hsa-miR-199b-5p	0.0161787	8.32E-04	2.598735
	hsa-let-7i	0.0161787	8.37E-04	2.073183
	hsa-miR-150	0.0175298	9.44E-04	3.28386
	hsa-miR-143	0.0175298	9.89E-04	2.713456
	hsa-miR-100	0.0180691	1.08E-03	2.544108
	hsa-miR-199a/b-3p	0.0194912	1.21E-03	2.445292
	hsa-miR-23a	0.0215577	1.63E-03	2.265833
	hsa-miR-125b	0.0233466	1.83E-03	2.846407
	hsa-miR-196a	0.0413646	4.15E-03	3.586379
Down-regulated	hsa-miR-630	0.0020383	2.80E-05	−4.218462
	hsa-miR-216a	0.0161787	7.81E-04	−2.636217
	hsa-miR-130b	0.0175298	9.84E-04	−2.612345
	hsa-miR-217	0.0211921	1.52E-03	−3.572319
	hsa-miR-655	0.0260153	2.08E-03	−2.216555
	hsa-miR-455-3p	0.0301557	2.86E-03	−2.038336
	hsa-miR-744	0.0492626	5.24E-03	−2.830606
	hsa-miR-220a	0.0542844	6.66E-03	−2.109385

### Enrichment analysis of the overlapping DEGs and miRNAs

**Figure 2:**
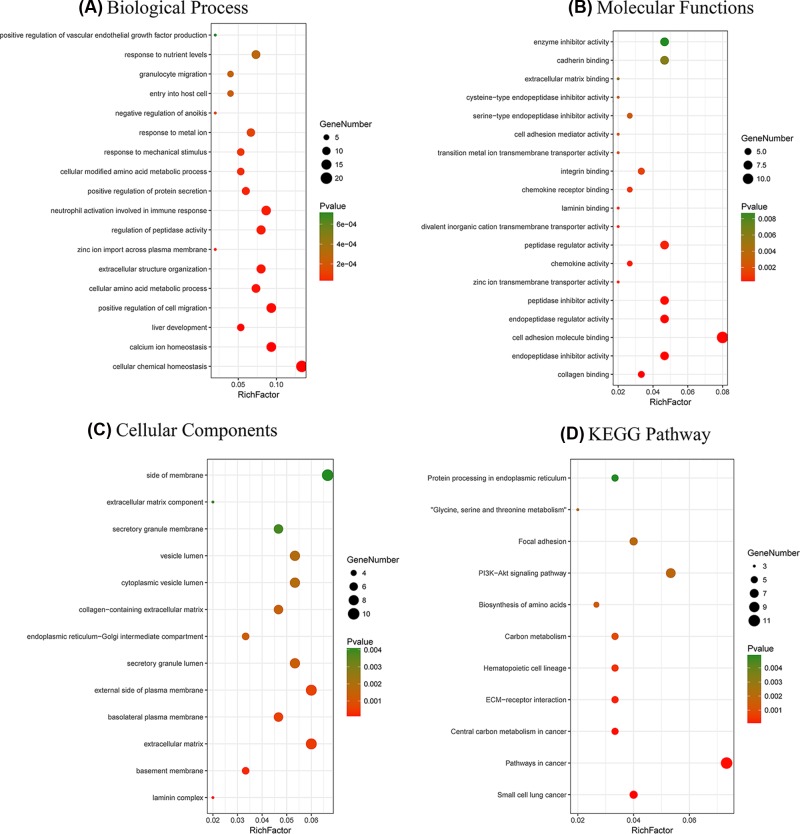
Functional and pathway enrichment analyses of the overlapping DEGs in PDAC (**A**) BP, (**B**) MF, (**C**) CC, (**D**) and KEGG pathway analyses. The x-axis represents the q value (−log10), and the y-axis represents the GO term. The GO terms were measured by the rich factor, q value and number of genes enriched. The greater the Rich factor is, the greater the degree of enrichment and the greater the p value [0, 1]. The brighter the color of red is, the more significant the term.

The BP analysis demonstrated that the overlapping DEGs were dramatically concentrated in cellular chemical homeostasis, carboxylic acid biosynthetic process, and the positive regulation of cell migration (Figure 2A). Regarding MF, the overlapping DEGs were significantly enriched in cell adhesion molecule binding (Figure 2B). Regarding CC, the overlapping DEGs were significantly enriched in the extracellular matrix and the external side of the plasma membrane (Figure 2C). Additionally, the KEGG analysis indicated that the overlapping DEGs were significantly enriched in pathways involved in cancer and the PI3K-Akt signaling pathway (Figure 2D).

**Figure 3:**
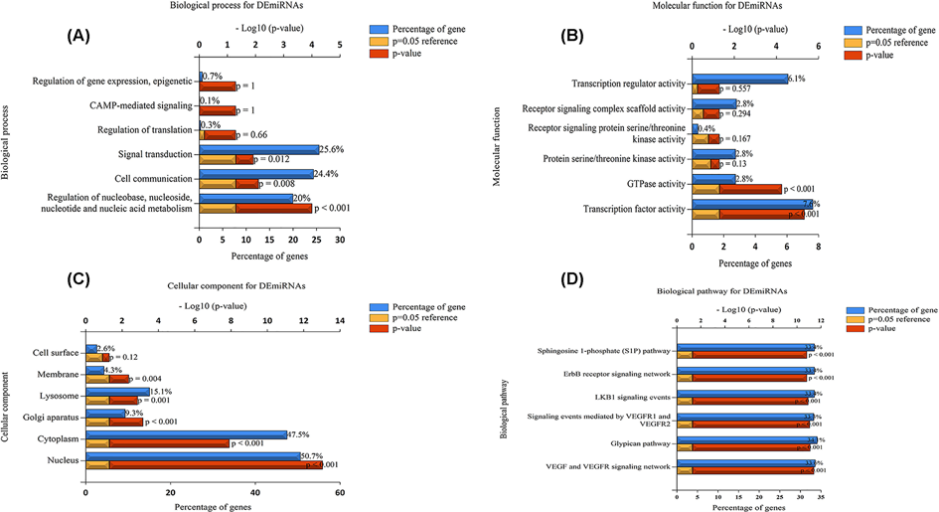
Functional and pathway enrichment analyses of the DEmiRNAs in PDAC (**A**) The BP analysis of miRNAs related to the DEGs, (**B**) the MF analysis of the miRNAs related to the DEGs, (**C**) the CC analysis of the miRNAs related to the DEGs, (**D**) and the biological pathway analysis of the miRNAs related to the DEGs. The upper x-axis represents the *P*-value (−log10), and the lower x-axis represents the percentage of genes (blue). The y-axis represents the GO term. Yellow represents the *P*-value equal to 0.05 as reference, and red represents the specific *P*-value. The longer the rectangular zone, the smaller the *P*-value is.

To improve our understanding of the biological information of the 32 DEmiRNAs in PDAC, we also performed GO annotation and KEGG pathway analyses. Regarding BP, the DEmiRNAs were significantly enriched in signal transduction and cell communication as well as the regulation of nucleobase, nucleoside, nucleotide, and nucleic acid metabolism (Figure 3A). Regarding MF, the DEmiRNAs were significantly enriched in transcription factor activity and GTPase activity (Figure 3B). In addition, the most enriched GO terms in CC were nucleus, cytoplasm, Golgi apparatus, and lysosome (Figure 3C). As shown in Figure 3D, the most enriched KEGG pathway terms for the DEmiRNAs were related to VEGF and the VEGFR signaling network, the glypican pathway, LKB1 signaling events, the ERBB receptor signaling network and the sphingosine 1-phosphate (S1P) pathway.

**Figure 4:**
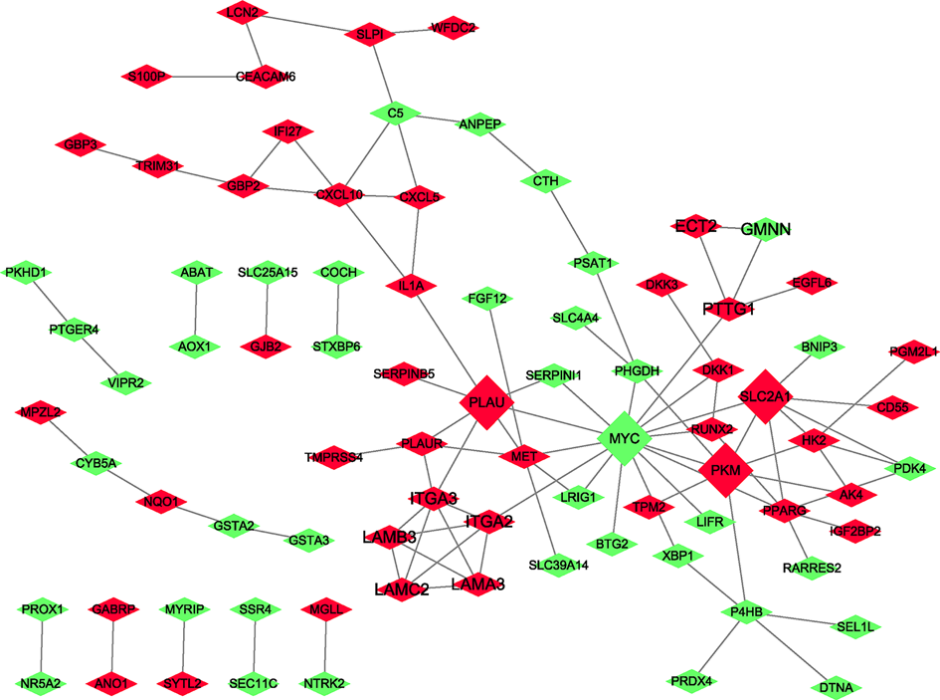
PPI network analysis of the overlapping DEGs Red nodes, up-regulated genes; green nodes, down-regulated genes.

To explore the interactions of the overlapping DEGs, a PPI network based on the STRING database was constructed. In total, the PPI network of the overlapping DEGs comprises 83 nodes and 99 edges (Figure 4). The more characteristic properties of node degree, the more significant influence it has on maintaining the stability of the PPI network. Seven nodes were identified as hub genes with a degree > 5, namely, MYC (degree = 15), SLC2A1 (degree = 7), PKM (degree = 7), PLAU (degree = 7), PPARG/PPARγ (degree = 6), MET (degree = 6), and ITGA3 (degree = 6).

### Survival analysis

**Figure 5:**
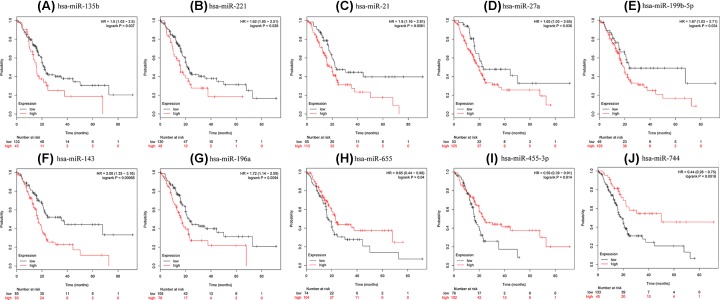
Overall survival analyses of differentially expressed microRNAs KM curves depicting OS for PDAC patients with high and low expression of (**A**) hsa-miR-135b, (**B**) hsa-miR-221, (**C**) hsa-miR-21, (**D**) hsa-miR-27a, (**E**) hsa-miR-199b-5p, (**F**) hsa-miR-143, (**G**) hsa-miR-196a, (**H**) hsa-miR-655, (**I**) hsa-miR-455-3p, and (**J**) hsa-miR-744.

**Figure 6:**
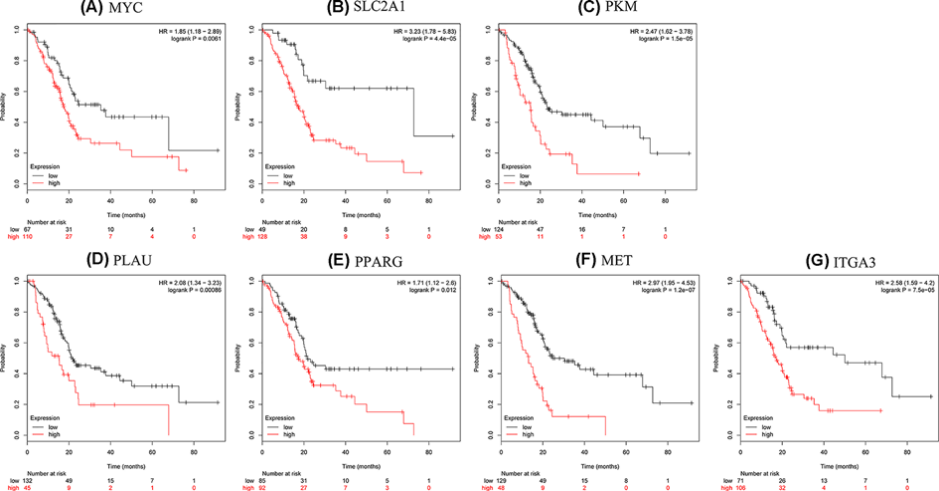
Prognostic value of seven DEGs in PC patients Prognostic value of (**A**) MYC (log-rank P-value = 0.0061), (**B**) SLC2A1 (log-rank P-value = 4.4e-05), (**C**) PKM (log-rank P-value = 1.5e-05), (**D**) PLAU (log-rank P-value = 0.00086), (**E**) PPARG (log-rank P-value = 0.012), (**F**) MET (log-rank P-value = 1.2e-07), and (**G**) ITGA3 (log-rank P-value = 7.5e-05).

KM plotter was employed to predict the prognostic values of the 32 identified DEmiRNAs and 7 hub genes. Among the DEmiRNAs examined, our results showed that the high expression of hsa-miR-221, hsa-miR-135b, hsa-miR-21, hsa-miR-199b-5p, hsa-miR-143, hsa-miR-27a, and hsa-miR-196a (*P*<0.05) was associated with worse OS for PDAC patients (Figure 5A–G). Additionally, low expression levels of hsa-miR-655, hsa-miR-455-3p, and hsa-miR-744 (*P<0.05*) were associated with poor OS for PDAC patients (Figure 5H–J). Similarly, the high expression of MYC, SLC2A1, PKM, PLAU, PPARG, MET, and ITGA3 may also be associated with the poor survival of patients with PDAC (*P*<0.05) (Figure 6).

### Transcriptional expression level of the hub genes and correlation analysis

**Figure 7:**
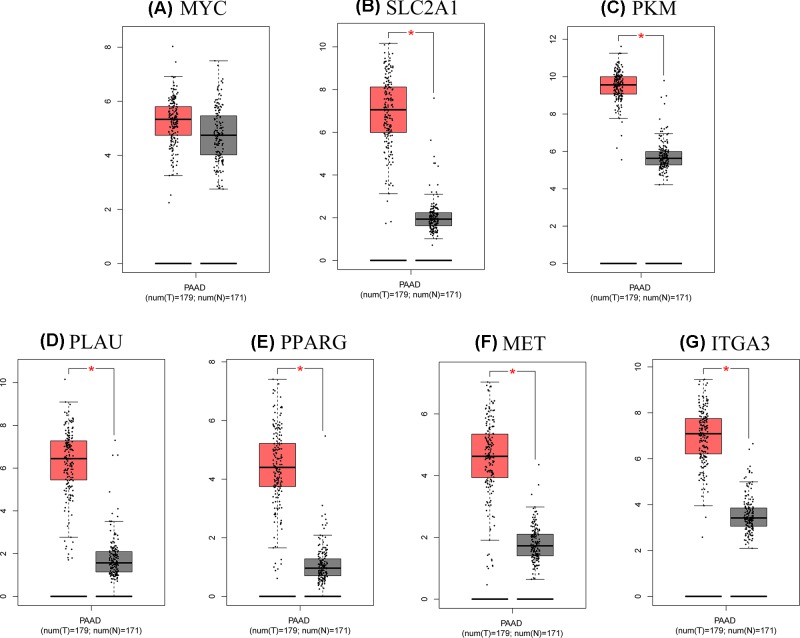
Expression levels of seven hub genes in human pancreatic adenocarcinoma (**A**) MYC; (**B**) SLC2A1; (**C**) PKM; (**D**) PLAU; (**E**) PPARG; (**F**) MET; and (**G**) ITGA3. The gray and red boxes represent normal and cancer tissues, respectively. The expression data are first log2(TPM+1) transformed for differential analysis and the log2FC is defined as median (Tumor, T)-median(Normal, N). *P<0.05, T vs N. Abbreviations: FC, fold change; TPM, transcript per kilobase million.

**Figure 8:**
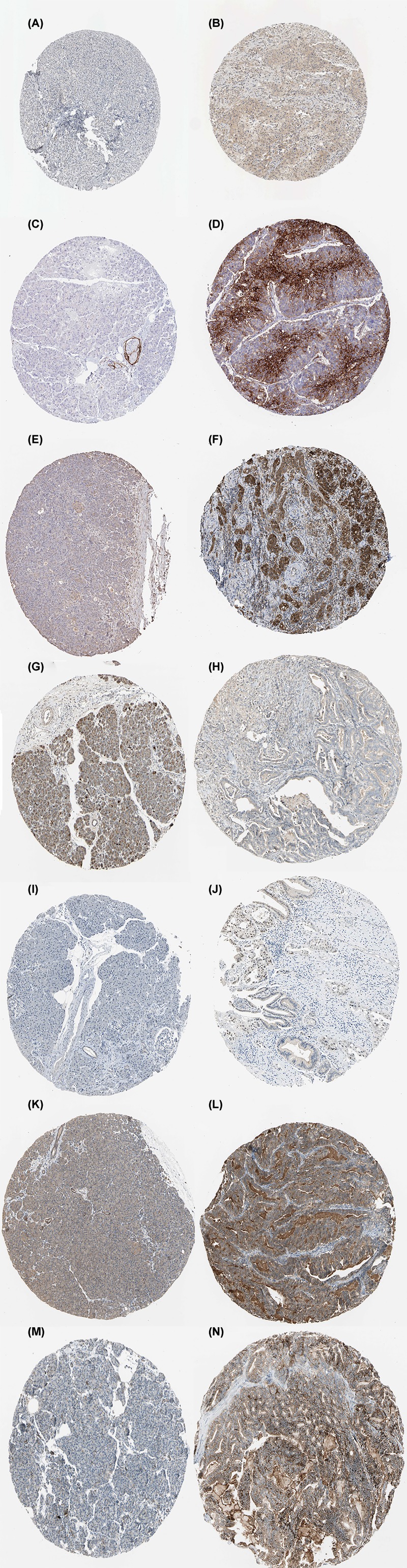
Immunohistochemistry of the five hub genes based on the HPA (**A**) Protein levels of MYC in normal tissue (staining: low; intensity: weak; quantity: >75%). (**B**) Protein levels of MYC in tumor tissue (staining: medium; intensity: moderate; quantity: 75–25%). (**C**) Protein levels of SLC2A1 in normal tissue (staining: not detected; intensity: negative; quantity: negative). (**D**) Protein levels of SLC2A1 in tumor tissue (staining: high; intensity: strong; quantity: 75–25%). (**E**) Protein levels of PKM in normal tissue (staining: low; intensity: weak; quantity: 75–25%). (**F**) Protein levels of PKM in tumor tissue (staining: high; intensity: strong; quantity: >75%). (**G**) Protein levels of PLAU in normal tissue (staining: not detected; intensity: negative; quantity: negative). (**H**) Protein levels of PLAU in tumor tissue (staining: low; intensity: weak; quantity: >75%). (**I**) Protein levels of PPARG in normal tissue (staining: not detected; intensity: negative; quantity: negative). (**J**) Protein levels of PPARG in tumor tissue (staining: medium; intensity: moderate; quantity: 75–25%). (**K**) Protein levels of MET in normal tissue (staining: medium; intensity: moderate; quantity: >75%). (**L**) Protein levels of MET in tumor tissue (staining: medium; intensity: moderate; quantity: >75%). (**M**) Protein levels of ITGA3 in normal tissue (staining: low; intensity: weak; quantity: >75%). (**N**) Protein levels of ITGA3 in tumor tissue (staining: high; intensity: strong; quantity: >75%).

**Figure 9:**
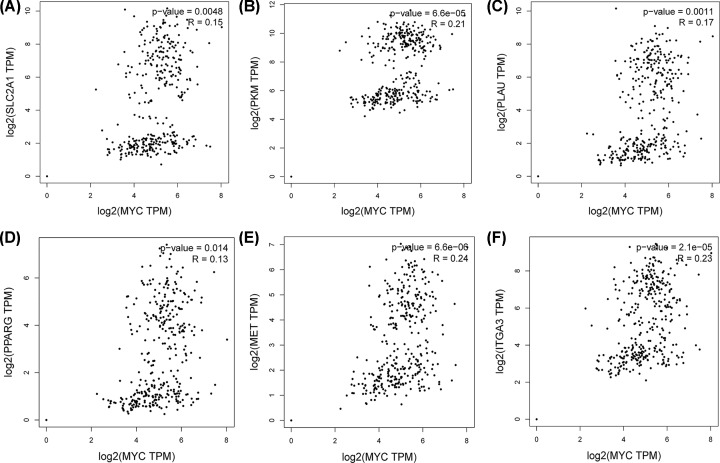
Correlation analysis of SLC2A1, PKM, PLAU, PPARG, MET, ITGA3, and MYC in pancreatic adenocarcinoma (**A**) SLC2A1, (**B**) PKM, (**C**) PLAU, (**D**) PPARG, (**E**) MET, and (**F**) ITGA3. TPM, transcript per million. The x-axis represents the TPM of the hub gene, MYC (log2), the y-axis represents the TPM of the hub genes SLC2A1, PKM, PLAU, PPARG, MET, and ITGA3 (log2). The expression levels of SLC2A1, PKM, PLAU, PPARG, MET, and ITGA3 were positively correlated with the MYC expression level.

As shown in Figure 7, the mRNA expression levels of seven genes were remarkably up-regulated in PDAC based on data from the GEPIA database. In addition, we used the ‘HPA’ to examine the protein expression levels of these hub genes and observed that the protein expression levels of the hub genes were noticeably up-regulated in tumor tissues compared with normal tissues (Figure 8). The increase in SLC2A1, PKM, PLAU, PPARG, MET, and ITGA3 was strongly associated with the increase in MYC in PDAC (Figure 9).

### miRNA–mRNA network

**Figure 10:**
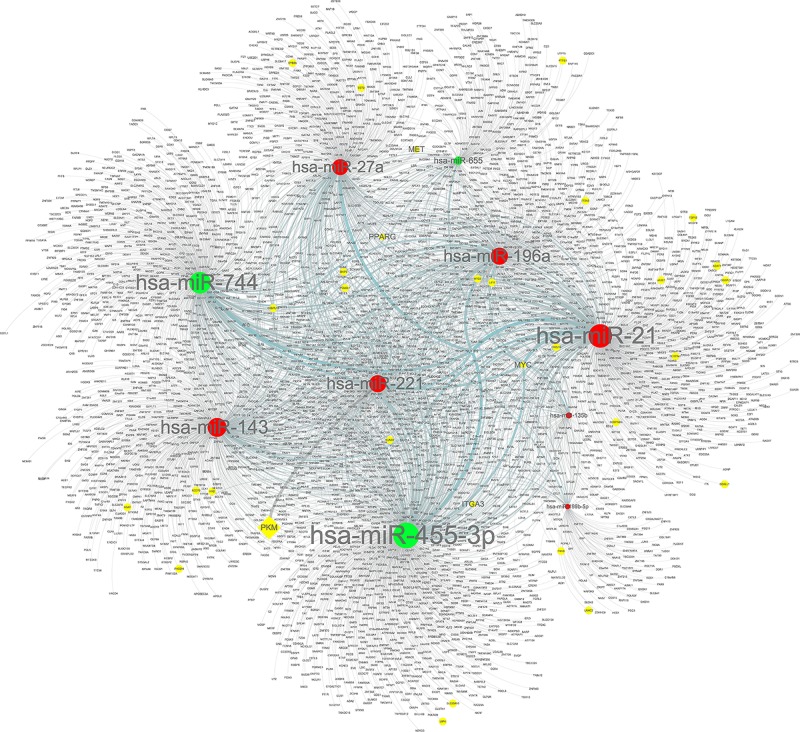
Regulatory network of the predicted genes and their target miRNAs Circle nodes indicate DEmiRNAs. Diamond nodes represent hub genes. Red nodes represent up-regulated DEmiRNAs, and green nodes represent down-regulated DEmiRNAs. Yellow nodes indicate DEGs.

The network of DEmiRNAs and predicted targets is presented in Figure 10. Notably, hsa-miR-135b targeted 137 genes, including MYC; hsa-miR-221 targeted 504 genes, including PKM; hsa-miR-21 targeted 686 genes, including MYC; hsa-miR-27a targeted 467 genes, including PPARG and MET; hsa-miR-199b-5p targeted 111 genes, including ITGA3; hsa-miR-143 targeted 595 genes; hsa-miR-196a targeted 507 genes, including MYC; hsa-miR-655 targeted 224 genes, including MYC; hsa-miR-455-3p targeted 784 genes, including MYC; and hsa-miR-744 targeted 665 genes, including MYC. As a group, a total of 31 of the 150 DEGs were contained in the miRNA–mRNA regulatory network (Figure 10 and Table 3).

**Table 3:** The interactive relationship between hub genes and miRNAs

Hub genes	DEmiRNAs
MYC	hsa-miR-196a, hsa-miR-21, hsa-miR-135b, hsa-miR-744, hsa-miR-455-3p, hsa-miR-655
SLC2A1	NA
PKM	hsa-miR-221
PLAU	NA
PPARG	hsa-miR-27a
MET	hsa-miR-27a
ITGA3	hsa-miR-199b-5p

**The intersection of DEGs in the miRNAs–mRNA regulatory network**

P4HB, RUNX2, MYC, BNIP3, PKM, PGM2L1, BTG2, SERPINB5, ACAT1, COBLL1, JADE1, SERPINI1, LIFR, OSBPL3, CXCL10, FGF12, ECT2, MET PPARG, OSBPL10, CYB5A, LAMC2, ITGA3, HK2, ABAT, REEP3, PHGDH, ITGA2, PTTG1, LIPH, SLC25A15.

## Discussion

MiRNAs can be used as biological markers for PCs [4,30], and PC is becoming a common malignant disease with an incidence that has continued to rise during the last few years [30,31]. Hence, specific biomarkers for accurate diagnostic tests and potential therapeutic targets for individualized therapy are yet to be fully determined in PDAC. In the current study, the mRNA and miRNA expression data obtained from the GEO database yielded an overall total of 150 genes (76 up-regulated and 74 down-regulated genes) and 32 miRNAs (24 up-regulated and 8 down-regulated miRNAs) that were differentially expressed in PDAC samples and normal tissues. The 150 DEGs were significantly enriched in cancer-related pathways. Moreover, 32 DEmiRNAs were significantly enriched in the glypican pathway. The glypicans comprise a family of glycosylphosphatidylinositol (GPI)-anchored heparan sulfate proteoglycans that has a remarkable effect on cell division and growth regulation. At present, six members (GPC1–6) of this family are recognized in vertebrates [32]. The glypican pathway, which was indicated in the KEGG pathway analysis, includes glypican 1 (GPC1, Entry: k08107), which promotes the pathogenesis of many human cancers [33]. Research shows that high levels of serum GPC1 indicate a poor prognosis in PDAC [34], suggesting that intervention of the glypican pathway may be a promising strategy for PDAC treatment. The increased expression of seven DEmiRNAs (miR-135b, miR-221, miR-21, miR-27a, miR-199b-5p, miR-143, and miR-196a) and seven hub genes (MYC, SLC2A1, PKM, PLAU, PPARG, MET, and ITGA3) were associated with worse OS; however, hsa-miR-655, hsa-miR-455-3p, and hsa-miR-744 had the opposite prognostic value. SLC2A1, PKM, PLAU, PPARG, MET, and ITGA3 were up-regulated in PDAC compared with normal tissues and were closely related to an increase in MYC.

MiRNAs can function as competing endogenous RNAs (ceRNAs), causing the degradation and/or inhibition of mRNA translation by binding to its 3′–untranslated region (3′-UTR) [35]. However, this function is not exactly consistent with the aforementioned pattern between the DEmiRNAs and hub genes, which may also play a prominent role in tumor onset and progression in PDAC. Munding et al. (2012) [36] showed that miR-135b was highly misregulated and was preferable to miR-196a and miR-210 as an indicator for identifying PDAC. Similarly, Han et al. (2017) [37] reported that miR-135b-5p was significantly up-regulated in PC tissue compared with adjacent tissue, and the overexpression of miR-135b-5p advanced proliferation and migration by decreasing the transcriptional expression level of SFRP4 by directly targeting its 3′-UTR. Wang et al. (2016) [38] showed that miR-221 bound to the 3′-UTR of endothelial nitric oxide synthase traffic inducer (NOSTRIN) and inhibited its expression, and up-regulated miR-221 expression correlated with unsatisfactory survival in PDAC, and another study demonstrated that miR-221 is an oncogenic miRNA that contributes to Capan-2 cell proliferation by regulating the PTEN-Akt pathway [39]. In addition, miR-21 promotes epidermal growth factor (EGF)-induced proliferation, inhibits cell apoptosis, and accelerates cell cycle progression by targeting the PI3K/AKT and MAPK/ERK signaling pathways [40]; it is also associated with poor OS and disease-free survival in PDAC [41]. Frampton et al. (2015) [42] identified three miRNAs (miR-21, miR-23a, and miR-27a) with high tumor expression, and the inhibition of miR-23a, miR-21, and miR-27a had promoting effects in decreasing the proliferation of PDAC cells in culture and the growth of xenograft tumors. A fibrotic tumor-associated stroma (TAS), which is believed to aggravate the clinical biological symptoms of PDAC, and miR-199a and miR-199b, which belong to the miR-199 family that is uniquely expressed in TAS cells, support the stromal miRNA feature, suggesting that the tumor microenvironment contributes to miRNA changes, which conversely have mechanical effects on the TAS [43]. Compared with normal tissues, miR-143 was up-regulated in both PDAC and PVAC tumor samples, which may reflect the histological features and biological behavior of different PCs [44]. Accumulating evidence indicates that the serum miR-196a expression level is able to predict the median survival time of PDAC patients [45,46]. The OS analysis of the up-regulated DEmiRNAs was consistent with the results of the present study. Epithelial-to-mesenchymal transition (EMT) has been reported to promote cancer progression. Harazono et al. (2013) [47] reported that the overexpression of miR-655, which is an EMT-suppressive miRNA that targets TGFBR2 and ZEB1, not only induced the up-regulation of E-cadherin and the downregulation of typical EMT inducers but also inhibited the invasion and migration of mesenchymal-like cancer cells. Furthermore, the upregulation of miR-655 inhibits cell invasion in esophageal squamous cell carcinoma [48], triple-negative breast cancer [49], and hepatocellular carcinoma [50]. MiRNA-455 (miR-455) is recognized as a broadly conserved noncoding RNA that has been implicated in various cancers. The function of miR-455 has been confirmed in prostate cancer [51], gastric cancer [52], and oral squamous cancer cells [53]. However, the potential effect of miR-455-3p has rarely been explored in PDAC. In the current study, the survival analysis demonstrated that the up-regulated expression of miR-455-3p in PDAC patients was associated with prolonged OS. miR-744 was found to be significantly up-regulated in PC and increased tumorigenicity by inhibiting multiple negative regulators of the Wnt/β-catenin pathway [54]. Furthermore, a high level of plasma miR-744 contributed to the poor progression-free survival of nonoperable PC patients [55]. Interestingly, contradictory results regarding the survival analysis in this study demonstrate that further investigation is needed to confirm the effect of miR-744.

MYC acts as a carcinogenic transcription factor that regulates at least 15% of genes involved in various cellular processes, including the differentiation, cell proliferation, apoptosis, and metabolism of PC [56,57]. Additionally, the KRAS/ERK/c-Myc axis is the major driving factor of tumorigenesis in PC. In normal cells, MYC is a transient protein that is present from 20 to 30 min and is uncontrollable in cancers. Hence, promoting the degradation of MYC is a promising treatment strategy for PDAC [57]. Metabolic deregulation is a hallmark of human cancers. SLC2A1, also named GLUT1, transports glucose and its analogs into cells. A previous study indicated that the up-regulation of SLC2A1 accelerated tumor cell proliferation and metastasis [58]. Pyruvate kinase muscle isozymes (PKMs) play an important role in adjusting metabolic changes during carcinogenesis. Cheng et al. (2018) [59] revealed that the increased expression of PKM1 and PKM2 affected cell invasion and migration in PDAC cells. Lu et al. (2018) [60] analyzed the related microarray datasets (GSE15471, GSE62452, GSE102238, GSE62165, and GSE16515) and identified the top 21 genes with high connectivity degrees; among them, PLAU is a plasminogen activator, ITGA3 plays a vital function in EMT, and the high expression of PLAU and ITGA3 is related to poor survival, consistent with the current research. The peroxisome proliferator activated receptors (PPARs), a member of the NR1 (thyroid-like) subfamily of NR, comprise PPARα (NR1C1), PPARβ/δ (NR1C2), and PPARγ (NR1C3). PPARγ, which is expressed in primary PDAC and has proven to be of clinical value when used as an independent prognostic factor, is closely related to a high clinical stage and has a close association with short OS [61]. MET, the receptor of hepatocyte growth factor, was identified as a cancer stem cell marker in PDAC. Tomihara et al. (2017) [62] also revealed that patients with high Met expression experienced a markedly shorter survival time than those with low Met expression, consistent with the current research. Altogether, these data support that the DEmiRNAs and hub genes shed light on the clinical utility of prognostic values in PDAC.

The combination of a multichip joint analysis and a bioinformatics analysis has a notable advantage in recognizing and confirming possible biomarkers and targets for malignant tumors, although there are some limitations to the current study. First, the number of samples in each microarray dataset was relatively small, which may have resulted in a few false-positive results, and second, these results were not validated; therefore, further mechanistic studies of larger samples are still needed in the future.

Taken together, the results of the bioinformatics analysis of four GEO microarray datasets of PC indicated that cancer-related pathways (KEGG Entry: k05200) and the glypican pathway (KEGG Entry: k08107) probably participate in the onset and development of PDAC. The high expression of miR-135b, miR-221, miR-21, miR-27a, miR-199b-5p, miR-143, miR-196a, MYC, SLC2A1, PKM, PLAU, PPARG, and ITGA3, as well as the low expression of miR-655, miR-455-3p, and miR-744, were observably related to unsatisfactory survival effects in patients with PDAC. Bioinformatics analyses revealed that different miRNAs exhibited diverse potential functions that were associated with the DEGs. However, further studies need to be implemented to explore the molecular mechanisms and biological functions of the miRNA/target gene axis and to estimate whether they can serve as novel potential biomarkers or therapeutic targets in PC patients.

## Abbreviations

ANXA2: annexin A2
BP: biological process
CC: cellular component
DEG: differentially expressed gene
DEmiRNA: differentially expressed microRNA
EMT: epithelial-to-mesenchymal transition
FC: fold-change
GEO: Gene Expression Omnibus
GEPIA: Gene Expression Profiling Interactive Analysis
GPC1: glypican 1
GTEx: Genotype-Tissue Expression
HPA: Human Protein Atlas
ITGA3: integrin subunit α 3
KEGG: Kyoto Encyclopedia of Genes and Genomes
KM: Kaplan–Meier
MET: MET proto-oncogene, receptor tyrosine kinase
MF: molecular function
miRNA: microRNA
mRNA: messenger RNA
MYC: V-myc avian myelocytomatosis viral oncogene homolog
OS: overall survival
PC: pancreatic cancer
PDAC: pancreatic ductal adenocarcinoma
PLAU: plasminogen activator, urokinase
PPARG: peroxisome proliferator activated receptor γ
PPI: protein–protein interaction
SLC2A1: solute carrier family 2 member 1
TAS: tumor-associated stroma
TCGA: The Cancer Genome Atlas

## References

[1] Zhao X., Ren Y., Cui N., Wang X. and Cui Y. (2018) Identification of key microRNAs and their targets in exosomes of pancreatic cancer using bioinformatics analysis. Medicine 97, e12632 10.1097/MD.0000000000012632 30278585 PMC6181532

[2] Zhang Z., Pan B., Lv S. (2018) Integrating microRNA expression profiling studies to systematically evaluate the diagnostic value of MicroRNAs in pancreatic cancer and validate their prognostic significance with the cancer genome atlas data. Cell. Physiol. Biochem. 49, 678–695 10.1159/000493033 30165365

[3] Shi G., Zhang J., Lu Z. (2017) A novel messenger RNA signature as a prognostic biomarker for predicting relapse in pancreatic ductal adenocarcinoma. Oncotarget 8, 110849–110860 10.18632/oncotarget.22861 29340021 PMC5762289

[4] Kojima M., Sudo H. and Kawauchi J. (2015) MicroRNA markers for the diagnosis of pancreatic and biliary-tract cancers. PLoS ONE 10, e0118220 10.1371/journal.pone.0118220 25706130 PMC4338196

[5] Yan W. and Yan L. (2015) Analysis of molecular pathways in pancreatic ductal adenocarcinomas with a bioinformatics approach. Asian Pac. J. Cancer Prev. 16, 2561–2567 10.7314/APJCP.2015.16.6.2561 25824797

[6] Shen Q., Yu M. and Jia J.K. (2018) Possible molecular markers for the diagnosis of pancreatic ductal adenocarcinoma. Med. Sci. Monit. 24, 2368–2376 10.12659/MSM.906313 29671412 PMC5928849

[7] Khan S. (2015) Cancer and the microbiome: potential applications as new tumor biomarker. Expert Rev. Anticancer Ther. 15, 317–330 10.1586/14737140.2015.992785 25495037

[8] Arthur J.C., Perez-Chanona E., Muhlbauer M. (2012) Intestinal inflammation targetscancer-inducing activity of the microbiota. Science 338, 120–123 10.1126/science.1224820 22903521 PMC3645302

[9] Khan S. (2017) Prediction of mycoplasma hominis proteins targeting in mitochondria and cytoplasm of host cells and their implication in prostate cancer etiology. Oncotarget 8, 30830–30843 10.18632/oncotarget.8306 27027344 PMC5458171

[10] Zakariah M. (2018) To decipher the mycoplasma hominis proteins targeting into the endoplasmic reticulum and their implications in prostate cancer etiology using next-generation sequencing data. Molecules 23, 994 10.3390/molecules23050994 PMC6099661 29695086

[11] Khan S., Imran A. and Khan A.A. (2016) Systems Biology approaches for the prediction of possible role of *Chlamydia pneumoniae* proteins in the etiology of lung cancer. PLoS ONE 11, e0148530 10.1371/journal.pone.0148530 26871581 PMC4752481

[12] Shahanavaj K. (2015) Potential role of *Escherichia coli* DNA mismatch repair proteins in colon cancer. Crit. Rev. Oncol. Hematol. 96, 475–482 10.1016/j.critrevonc.2015.05.002 26014615

[13] Su Q., Zhu E.C., Qu Y.L. (2018) Serum level of co-expressed hub miRNAs as diagnostic and prognostic biomarkers for pancreatic ductal adenocarcinoma. J Cancer 9, 3991–3999 10.7150/jca.27697 30410604 PMC6218787

[14] Lv F., Zheng K., Yu J. (2018) MicroRNA-661 expression is upregulated in pancreatic ductal adenocarcinoma and promotes cell proliferation. Oncol. Lett. 16, 6293–6298 30405764 10.3892/ol.2018.9454 PMC6202501

[15] Wei D.M., Dang Y.W., Feng Z.B. (2018) Biological effect and mechanism of the miR-23b-3p/ANXA2 axis in pancreatic ductal adenocarcinoma. Cell. Physiol. Biochem. 50, 823–840 10.1159/000494468 30355917

[16] Alshamsana A.W.S. (2017) Prediction of *Chlamydia pneumoniae* protein localization in host mitochondria and cytoplasm and possible involvements in lung cancer etiology: a computational approach. Saudi Pharm. J. 25, 1151–1157 10.1016/j.jsps.2017.05.007 30166903 PMC6111117

[17] Khan S. (2016) Computational prediction of *Mycoplasma hominis* proteins targeting in nucleus of host cell and their implication in prostate cancer etiology. Tumour Biol. 37, 10805–10813 10.1007/s13277-016-4970-9 26874727

[18] Yi H.C., Liu Y.L., You P., Pan J.S., Zhou J.Y., Liu Z.J. (2015) Overexpression of DEK gene is correlated with poor prognosis in hepatocellular carcinoma. Mol. Med. Rep. 11, 1318 10.3892/mmr.2014.2781 25351213

[19] Barrett T., Wilhite S.E., Ledoux P., Evangelista C., Kim I.F., Tomashevsky M. (2013) NCBI GEO: archive for functional genomics data sets-update. Nucleic Acids Res. 41, D991–D995 10.1093/nar/gks1193 23193258 PMC3531084

[20] Frampton A.E., Castellano L., Colombo T., Giovannetti E. (2014) MicroRNAs cooperatively inhibit a network of tumor suppressor genes to promote pancreatic tumor growth and progression. Gastroenterology 146, 268.e18–277.e18 10.1053/j.gastro.2013.10.010 24120476

[21] Park M., Kim M., Hwang D., Park M. (2014) Characterization of gene expression and activated signaling pathways in solid-pseudopapillary neoplasm of pancreas. Mod. Pathol. 27, 580–593 10.1038/modpathol.2013.154 24072181

[22] Lunardi S., Jamieson N.B., Lim S.Y., Griffiths K.L. (2014) IP-10/CXCL10 induction in human pancreatic cancer stroma influences lymphocytes recruitment and correlates with poor survival. Oncotarget 5, 11064–11080 10.18632/oncotarget.2519 25415223 PMC4294325

[23] Tripathi S., Pohl M.O., Zhou Y., Rodriguez-Frandsen A., Wang G., Stein D.A. (2015) Meta-and orthogonal integration of influenza “OMICs” data defines a role for UBR4 in virus budding. Cell Host Microbe 18, 723–735 10.1016/j.chom.2015.11.002 26651948 PMC4829074

[24] Pathan M., Keerthikumar S., Chisanga D. (2017) A novel community driven software for functional enrichment analysis of extracellular. J. Extracell. Vesicles 6, 1321455 10.1080/20013078.2017.1321455 28717418 PMC5505018

[25] Shen S., Kong J., Qiu Y. (2019) Identification of core genes and outcomes in hepatocellular carcinoma by bioinformatics analysis. J. Cell. Biochem. 120, 10069–10081 10.1002/jcb.28290, 30525236

[26] Lanczky A., Nagy A., Bottai G., Munkacsy G., Paladini L., Szabo A. (2016) miRpower: a web-tool to validate survival-associated miRNAs utilizing expression data from 2,178 breast cancer patients. Breast Cancer Res. Treat. 160, 439–446 10.1007/s10549-016-4013-7 27744485

[27] Tang Z., Li C. and Kang B. (2017) GEPIA: a web server for cancer and normal gene expression profiling and interactive analyses. Nucleic Acids Res. 45, W98–W102 10.1093/nar/gkx247 28407145 PMC5570223

[28] Uhlén M., Fagerberg L., Hallström B.M. (2015) Proteomics. Tissue-based map of the human proteome. Science 347, 1260419 10.1126/science.1260419 25613900

[29] Chou C.H., Shrestha S. and Yang C.D. (2018) miRTarBase update 2018: a resource for experimentally validated microRNA-target interactions. Nucleic Acids Res. 46, D296–D302 10.1093/nar/gkx1067 29126174 PMC5753222

[30] Liu B., Yang H. and Taher L. (2018) Identification of prognostic biomarkers by combined mRNA and miRNA expression microarray analysis in pancreatic cancer. Transl. Oncol. 11, 700–714 10.1016/j.tranon.2018.03.003 29631214 PMC6154866

[31] Malvezzi M., Carioli G., Bertuccio P., Boffetta P., Levi F., La Vecchia C. (2017) European cancer mortality predictions for the year 2017, with focus on lung cancer. Ann. Oncol. 28, 1117–1123 10.1093/annonc/mdx033 28327906

[32] Szpirer C., Szpirer J., Riviere M., Van Vooren P., Veugelers M. and David G. (2001) Mapping of the rat glypican genes. Cytogenet. Cell Genet. 93, 83–86 10.1159/000056954 11474185

[33] Li J., Chen Y., Zhan C. (2019) Glypican-1 promotes tumorigenesis by regulating the PTEN/Akt/β-catenin signaling pathway in esophageal squamous cell carcinoma. Dig Dis. Sci. 64, 1493–1502 10.1007/s10620-019-5461-9 30730015

[34] Zhou C.-Y., Dong Y.-P., Sun X. (2018) High levels of serum glypican-1 indicate poor prognosis in pancreatic ductal adenocarcinoma. Cancer Med. 7, 5525–5533 10.1002/cam4.1833 30358133 PMC6246926

[35] Liang L., Wei D.-M., Li J.-J. (2017) Prognostic microRNAs and their potential molecular mechanism in pancreatic cancer: a study based on The Cancer Genome Atlas and bioinformatics investigation. Mol. Med. Rep. 17, 939–951 29115476 10.3892/mmr.2017.7945 PMC5780175

[36] Munding J.B., Adai A.T. and Maghnouj A. (2012) Global microRNA expression profiling of microdissected tissues identifies miR-135b as a novel biomarker for pancreatic ductal adenocarcinoma. Int. J. Cancer 131, E86–E95 10.1002/ijc.26466 21953293

[37] Han X., Saiyin H. and Zhao J. (2017) Overexpression of miR-135b-5p promotes unfavorable clinical characteristics and poor prognosis via the repression of SFRP4 in pancreatic cancer. Oncotarget 8, 62195–62207 28977937 10.18632/oncotarget.19150 PMC5617497

[38] Wang J., Yang S. and He P. (2016) Endothelial nitric oxide synthase traffic inducer (NOSTRIN) is a negative regulator of disease aggressiveness in pancreatic cancer. Clin. Cancer Res. 22, 5992–6001 10.1158/1078-0432.CCR-16-0511 27401251 PMC5161709

[39] Yang W., Yang Y., Xia L. (2016) MiR-221 promotes Capan-2 pancreatic ductal adenocarcinoma cells proliferation by targeting PTEN-Akt. Cell. Physiol. Biochem. 38, 2366–2374 10.1159/000445589 27230035

[40] Zhang L., Yao J., Li W. (2017) Micro-RNA-21 regulates cancer-associated fibroblast-mediated frug resistance in pancreatic cancer. Oncol. Res. 26, 827–836 10.3727/096504017X14934840662335 28477403 PMC7844724

[41] Guraya S. (2018) Prognostic significance of circulating microRNA-21 expression in esophageal, pancreatic and colorectal cancers; a systematic review and meta-analysis. Int. J. Surg. 60, 41–47 10.1016/j.ijsu.2018.10.030 30336280

[42] Frampton A.E., Castellano L. and Colombo T. (2015) Integrated molecular analysis to investigate the role of microRNAs in pancreatic tumour growth and progression. Lancet 385, S37 10.1016/S0140-6736(15)60352-X 26312859

[43] Han S., Gonzalo D.H., Feely M. (2017) The pancreatic tumor microenvironment drives changes in miRNA expression that promote cytokine production and inhibit migration by the tumor associated stroma. Oncotarget 8, 54054–54067 28903323 10.18632/oncotarget.10722 PMC5589562

[44] Tavano F., Mola F.F.D. and Piepoli A. (2012) Changes in miR-143 and miR-21 expression and clinicopathological correlations in pancreatic cancers. Pancreas 41, 1280–1284 10.1097/MPA.0b013e31824c11f4 22836856

[45] Xu Y.F., Hannafon B.N. and Zhao Y.D. (2017) Plasma exosome miR-196a and miR-1246 are potential indicators of localized pancreatic cancer. Oncotarget 8, 77028–77040 29100367 10.18632/oncotarget.20332 PMC5652761

[46] Yu Q., Xu C., Yuan W. (2017) Evaluation of plasma microRNAs as diagnostic and prognostic biomarkers in pancreatic adenocarcinoma: miR-196a and miR-210 could be negative and positive prognostic markers, respectively. Biomed. Res. Int. 2017, 6495867 10.1155/2017/6495867 28466015 PMC5390608

[47] Harazono Y., Muramatsu T. and Endo H. (2013) miR-655 Is an EMT-suppressive microRNA targeting ZEB1 and TGFBR2. PLoS ONE 8, e62757 10.1371/journal.pone.0062757 23690952 PMC3653886

[48] Wang Y., Zang W. and Du Y. (2013) Mir-655 up-regulation suppresses cell invasion by targeting pituitary tumor-transforming gene-1 in esophageal squamous cell carcinoma. J. Transl. Med. 11, 301 10.1186/1479-5876-11-301 24314023 PMC4029436

[49] Lv Z.-D., Kong B., Liu X.-P. (2016) MiR-655 suppresses epithelial-to-mesenchymal transition by targeting Prrx1 in triple-negative breast cancer. J. Cell. Mol. Med. 20, 864–873 10.1111/jcmm.12770 26820102 PMC4831358

[50] Wu G., Zheng K. and Xia S. (2016) MicroRNA-655-3p functions as a tumor suppressor by regulating ADAM10 and β-catenin pathway in hepatocellular carcinoma. J. Exp. Clin. Cancer Res. 35, 1–12 27259866 10.1186/s13046-016-0368-1 PMC4893252

[51] Zhao Y., Yan M., Yun Y. (2017) MicroRNA-455-3p functions as a tumor suppressor by targeting eIF4E in prostate cancer. Oncol. Rep. 37, 2449–2458 10.3892/or.2017.5502 28350134

[52] Liu J., Zhang J. and Li Y. (2016) MiR-455-5p acts as a novel tumor suppressor in gastric cancer by down-regulating RAB18. Gene 592, 308–315 10.1016/j.gene.2016.07.034 27451075

[53] Cheng C.M., Shiah S.G., Huang C.C., Hsiao J.R. and Chang J.Y. (2016) Upregulationof miR-455-5p by the TGF-b-SMAD signalling axis promotes the proliferation of oral squamous cancer cells by targeting UBE2B. J. Pathol. 240, 38–49 10.1002/path.4752 27235675

[54] Zhou W., Li Y. and Gou S. (2015) MiR-744 increases tumorigenicity of pancreatic cancer by activating Wnt/β-catenin pathway. Oncotarget 6, 37557–37569 10.18632/oncotarget.5317 26485754 PMC4741948

[55] Mahito M., Shuhei K. and Daisuke I. (2015) Plasma microRNA profiles: identification of miR-744 as a novel diagnostic and prognostic biomarker in pancreatic cancer. Br. J. Cancer 113, 1467–1476 10.1038/bjc.2015.366 26505678 PMC4815891

[56] Hessmann E., Schneider G. and Ellenrieder V. (2015) MYC in pancreatic cancer: novel mechanistic insights and their translation into therapeutic strategies. Oncogene 35, 1609–1618 10.1038/onc.2015.216 26119937

[57] Wirth M., Mahboobi S., Kramer O.H. and Schneider G. (2016) Concepts to target MYC in pancreatic cancer. Mol. Cancer Ther. 15, 1792–1798 10.1158/1535-7163.MCT-16-0050 27406986

[58] Yan S., Wang Y., Chen M., Li G. and Fan J. (2015) Deregulated SLC2A1 promotes tumor cell proliferation and metastasis in gastric cancer. Int. J. Mol. Sci. 16, 16144–16157 10.3390/ijms160716144 26193257 PMC4519943

[59] Cheng T.Y., Yang Y.C. and Wang H.P. (2018) Pyruvate kinase M2 promotes pancreatic ductal adenocarcinoma invasion and metastasis through phosphorylation and stabilization of PAK2 protein. Oncogene 37, 1730–1742 10.1038/s41388-017-0086-y 29335522

[60] Lu Y., Li C., Chen H. and Zhong W. (2018) Identification of hub genes and analysis of prognostic values in pancreatic ductal adenocarcinoma by integrated bioinformatics methods. Mol. Biol. Rep., 45, 1799–1807 10.1007/s11033-018-4325-2 30173393

[61] Polvani S., Tarocchi M. and Tempesti S. (2014) Nuclear receptors and pathogenesis of pancreatic cancer. World J. Gastroenterol. 20, 12062–12081 10.3748/wjg.v20.i34.12062 25232244 PMC4161795

[62] Tomihara H., Yamada D. and Eguchi H. (2017) MiR-181b-5p, ETS1 and c-Met pathway exacerbates the prognosis of pancreatic ductal adenocarcinoma after radiation therapy. Cancer Sci. 108, 398–407 10.1111/cas.13159 28064436 PMC5378264

